# The ride of the parasite: a 100-million-year old mantis lacewing larva captured while mounting its spider host

**DOI:** 10.1186/s40851-018-0116-9

**Published:** 2018-12-22

**Authors:** Joachim T. Haug, Patrick Müller, Carolin Haug

**Affiliations:** 1 Ludwig Maximilians University Munich, Biocenter, Großhaderner Str. 2, 82152, Planegg-Martinsried, Germany; 2 GeoBio-Center at LMU, Richard-Wagner-Str. 10, 80333 Munich, Germany; 3 Käshofen, Germany

**Keywords:** Mantispidae, Neuroptera, Hypermetaboly, Burmese amber, Palaeo-parasitism

## Abstract

**Background:**

Adult mantis lacewings, neuropteran holometabolan insects of the group Mantispidae, possess anterior walking legs transformed into prey-catching grasping appendages reminiscent of those of praying mantises. While adult mantis lacewings are hence active “wait-and-catch” predators, the larvae of many mantis lacewings have a quite different biology: first-stage larvae seek out female spiders, mount them, and either wait until the spider has produced an egg sac or, in some cases, choose a female already bearing an egg sac. The larva then enters the egg sac and feeds on the eggs. While first stage larvae are highly mobile with comparably long legs and a certain degree of dorso-ventral flattening (“campodeiform”), larval stages two and three are almost immobile, grub-like, and simply remain within the egg sac. Fossils of mantis lacewings are relatively rare, fossils of larval mantis lacewings are even rarer; only a single larva sitting on a juvenile spider has been described from ca. 50 million year old Baltic amber.

**Results:**

Here we describe a second occurrence of a larval mantis lacewing from significantly older Burmese amber, about 100 million years old. The specimen is preserved in a position right at the leg of a spider, similar to modern-day larvae that are about to mount their prospective host. The claws of the larva can be seen to grab around the leg of the spider.

**Conclusions:**

We discuss how reliable these fossils are as indicators of palaeo-parasitism, and in which aspects the behaviour of mantis lacewing larvae in general indeed represents parasitism. While the specimen appears to be about to board the spider, it may not necessarily represent a parasite in the strict sense. Evaluating the actual ecological role of a fossil heavily depends on comparison to modern forms, and not all modern-day larvae of Mantispidae are parasites. We therefore provide a closer look into the known feeding habits of modern mantis lacewing larvae.

## Background

Of the neuropteran insects, or lacewings, green lacewings are the most familiar, while other groups within Neuroptera are less well known, but can be quite fascinating nonetheless. Adult representatives of Mantispidae, or mantis lacewings, possess a pair of highly specialised first thoracic appendages, i.e., these have become transformed into prominent subchelate grasping appendages with which they catch prey. As their name suggests, these appendages resemble the grasping legs of praying mantises (Mantodea).

While adult mantis lacewings are highly specialised, their larval stages are even more unusual. Most neuropteran insects develop through three larval stages, which are subsimilar. This is quite different in mantis lacewings. The first larval stage resembles the larval stages of many other neuropterans; it is a fully mobile, holometabolan larva, with prominent legs, slightly dorso-ventrally compressed (‘campodeiform’) body, and its mandibles and maxillae form a pair of piercing-sucking mouth parts. The next two stages are usually described as maggot-like [1], but could also be called grub-like; the body lacks most sclerotisations, has a more circular diameter, but is rather wide in the middle; the legs are very short and hardly visible. Hence, the first larval stage shows more characteristics of the later adult than the second and third larval stage. This type of ‘developmental detour’ is generally referred to as ‘hypermetamorphosis’ [see 1 and references therein]. Other authors refer to similar kinds of development as ‘hypermetabolous metamorphosis’ or ‘hypermetaboly’ [2, 3].

The first stage larvae of many mantis lacewing species, those of Mantispinae, seek for a spider female, mount it and finally enter an egg sac produced by the spider and feed on the eggs [4, 5]. The two immobile stages stay in the egg sac on the same female spider before moulting into the pupa as soon as the energy resource, i.e., the eggs, is exploited (yet in some cases individual eggs may survive; [6]). This makes the larval forms of Mantispinae quite unusual among lacewing larvae, which are usually all active predators. Other groups of Mantispidae also do not appear to be active predators in their larval phase. At least one species of Symphrasinae appears to be a parasitoid of crabronid wasps [7]. Other species of Symphrasinae, Drepanicinae and Calomantispinae appear to have a broader food spectrum and may indeed be predators ([8] and references therein).

About 350 species of mantis lacewings are known in the modern fauna. Fossil mantis lacewings are comparatively rare [1]. Fossils of mantis lacewing larvae are even rarer. In fact, so far only a single specimen has been described from 50-million-year-old Baltic amber [1]. The small larva was found embedded into an amber piece together with a juvenile spider and has its head deeply concealed between the prosoma (functional head) and the opisthosoma (trunk) of the spider.

We here report a second fossil of a mantis lacewing larva also found in association with a spider, in fact with two spiders. The larva with the associated spiders is significantly older than the previously known one as it is enclosed in Burmese amber. With this, it represents the currently oldest report of a mantis lacewing larva. We discuss in how far this case is an example for palaeo-parasitism, in how far mantis lacewing larvae are parasites, and if our new finding indicates the occurrence of hypermetaboly in the Cretaceous.

## Materials and methods

### Materials

The single amber piece in the center of this study comes from the about 100-million-year-old Burmese deposits, more exactly the Hukawng Valley, Kachin State, Myanmar [9]. It has been bought by one of the authors (PM) and is currently part of his private collection under the repository number BUB 3068. It is now part of the collection of the Staatliches Museum für Naturkunde Stuttgart (collection number SMNS-P-BU-338).

The raw amber piece was first cut with a Dremel 3000. Afterwards it was polished with wet sandpaper, first grade 200 and then subsequently grades 600, 1000 and 5000. The final polish was performed with Sidol metal polish.

### Documentation methods

The specimen was documented with composite imaging under different white light conditions. The images were recorded with a Keyence VHX-6000 microscope equipped with a 20–2000× objective, either under ring illumination or under coaxial cross-polarised illumination. To achieve an optimal result, some images were recorded with different exposure times (high dynamic range, HDR).

Each image detail was documented as a stack, with the single images of the stack (frames) being recorded in different focal levels in the z-axis to overcome limitations in depth of field. The frames of each stack were fused to achieve an entirely sharp image detail. Several adjacent stacks were recorded in x-y axis to overcome limitations in the field of view. All image details were stitched to a final panorama image [10, 11].

Drawings of the specimen and of comparative material were prepared in Adobe Illustrator CS2. Colour markings of specific structures was performed in Adobe Photoshop CS2.

## Results

### Description of the amber piece

**Fig. 1:**
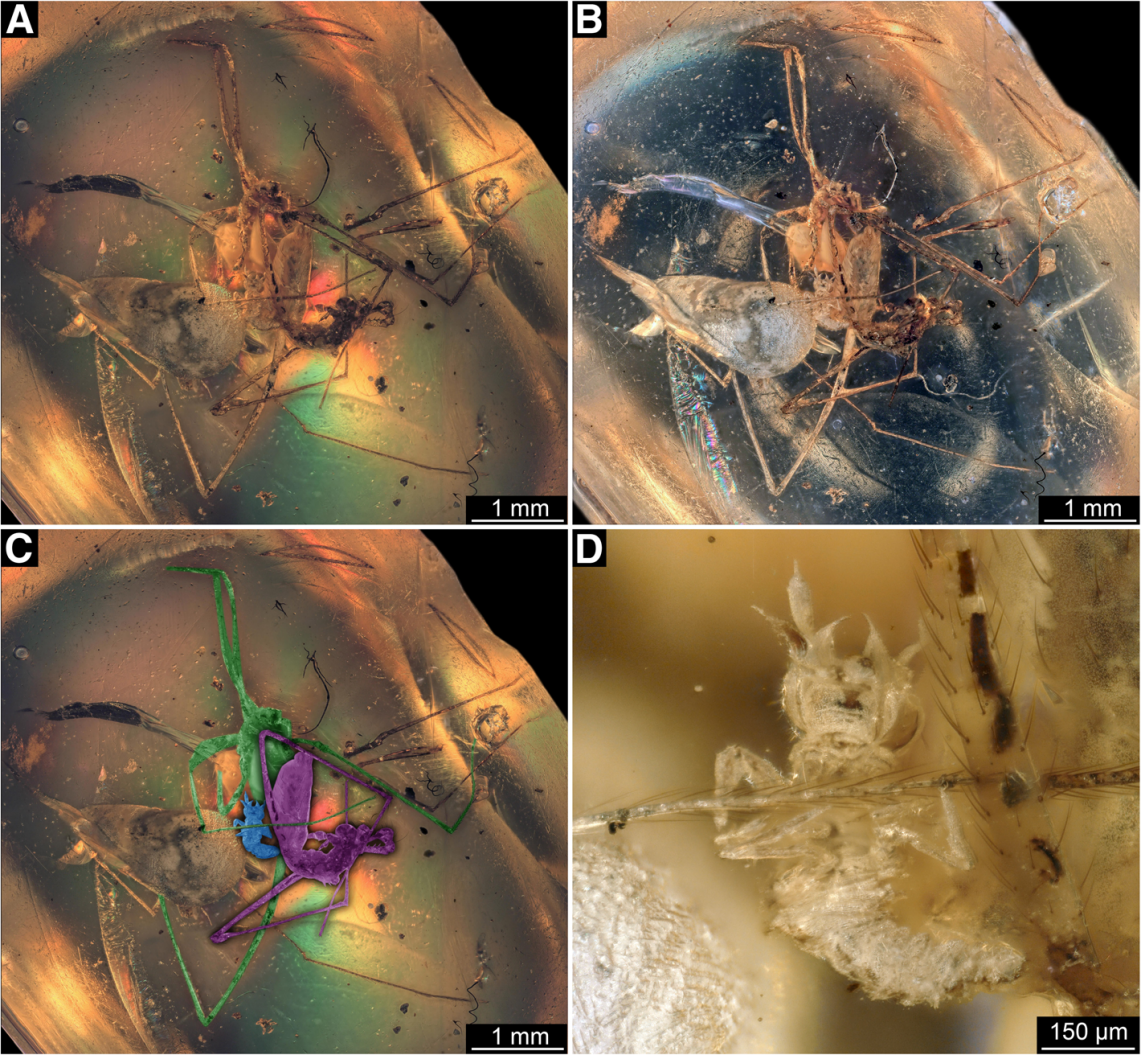
Overview of the amber piece with the mantis lacewing larva. **a**–**c**. Overview images. **a** Under coaxial cross-polarised light. **b** Under low-angle reflective ring illumination. **c** Colour-marked version of A; male spider (*Priscaleclercera*) in purple; female spider (*Burmorchestina*) in green; neuropteran larva in blue. **d** Close-up on larva; coaxial cross-polarised light, single exposure time

The amber piece contains (at least) three euarthropod inclusions: Two spiders and a small insect larva (Fig. 1a–c). One of the spiders could be identified as male of the group *Priscaleclercera* (Psilodercidae; det. Jörg Wunderlich, 2018) [12]. The other spider is a female of the group *Burmorchestina* (Oecobiidae; det. Jörg Wunderlich, 2018) [12]. Both spiders are positioned close to each other, and following the legs carefully is necessary to identify which leg belongs to which spider specimen (Fig. 1c). Some of the legs also appear detached. It is nonetheless apparent that a small insect larva is attached to one of the anterior walking legs of the female spider(Fig. 1d). Head structures of the larva indicate that it is a neuropteran insect.

### Description of the larval neuropteran specimen

#### Overall habitus

**Fig. 2:**
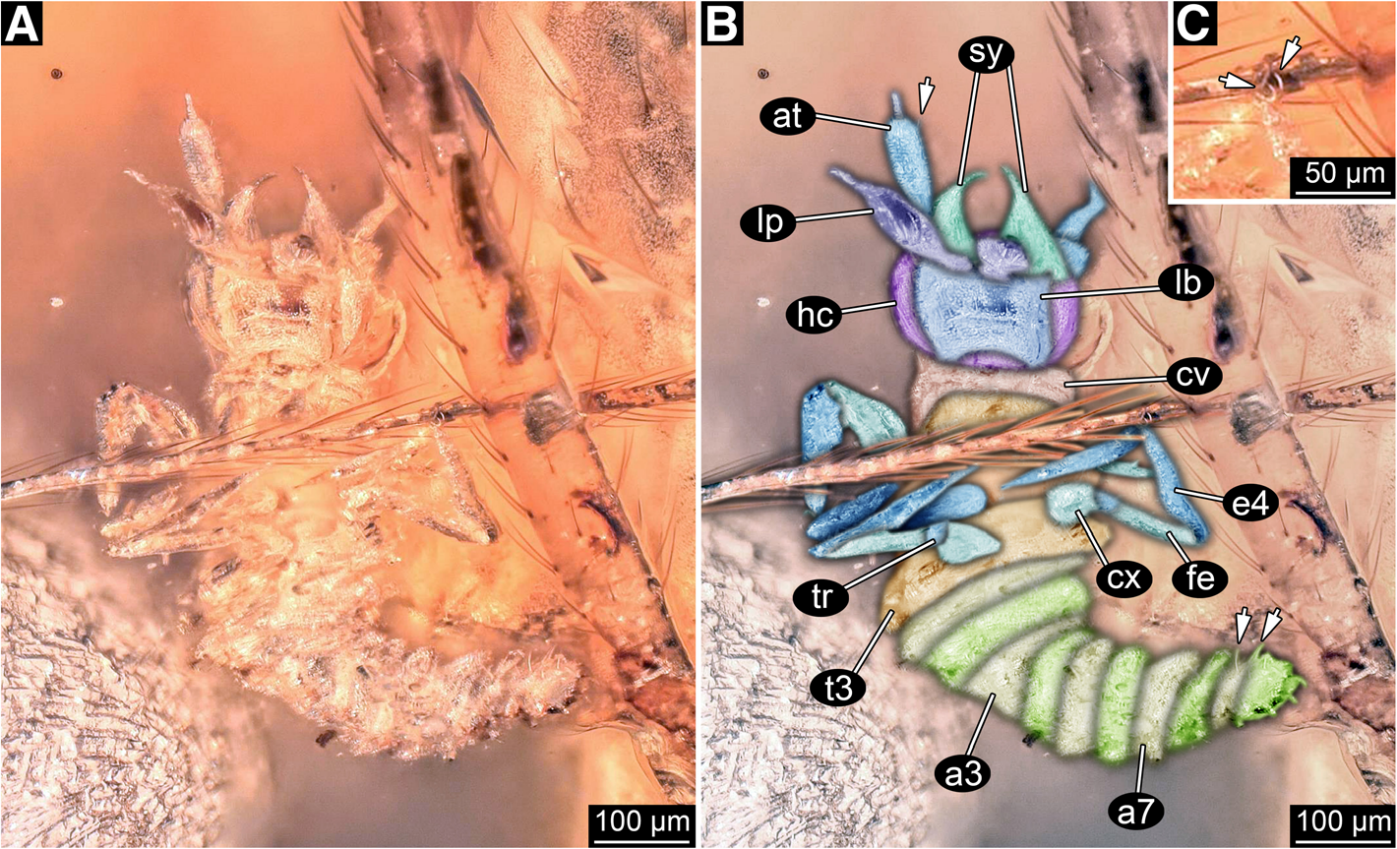
Details of the mantis lacewing larva. **a** Close-up of larva; coaxial cross-polarised light, HDR. **b** Colour-marked version of A; arrows mark some of the setae. **c** Close-up of claws (arrows). Abbreviations: a3 = abdominal segment 3 (post-ocular segment 11); a7 = abdominal segment 7 (post-ocular segment 15); at = antenna (antennula in euarthropod terminology); cv = cervix (neck region); cx = coxa (appendage element 1); e4 = appendage element 4 (tibia+tarsus?); fe = femur (appendage element 3); hc = head capsule; lb. = labium; lp = labial palp; sy = mandibular-maxillary stylet; t3 = thoracic segment 3 (post-ocular segment 8); tr = trochanter (appendage element 2)

Entire body length without appendages ca. 750 μm. Body organised into (presumably) 20 segments (Fig. 2a, b). Segments organised into three major functional units, or tagmata. First six segments, ocular segments and post-ocular segments 1–5, form head tagma with distinct head capsule. Post-ocular segments 6–19 form the trunk. Trunk further differentiated; anterior three trunk segments (post-ocular segments 6–8) with prominent appendages on the ventral side. This tagma is termed the thorax, the appendages thoracopods. Posterior trunk segments (post-ocular segments 9–19) forming a tagma, which is termed the abdomen (insect-type abdomen; not corresponding to that of other crustaceans). Most of the surface appears grainy.

#### Head

Head capsule largely obscured by ventral structures. No structures of ocular segments apparent due to orientation of the fossil. Post-ocular segment 1 recognisable by its appendages, the antennae (antennulae in neutral arthropod terminology). Most likely composed of several elements, yet due to orientation only one prominent element apparent, most likely element 2 or 3 (counted from proximal). The element is roughly club-shaped, widening distally. A single small seta is apparent, most likely more such setae were originally present. Distally a thin structure articulates with the club-shaped element. This represents either an additional element or a very thick seta. Post-ocular segment 2 (intercalary segment) is not externally recognisable.

Post-ocular segments 3 and 4 recognisable by a pair of prominent structures formed by the appendages of these segments. Appendages of post-ocular segment 3 (mandibles) and 4 (maxillae; maxillulae in more neutral arthropod terminology) seem to form a pair of stylets. An individual stylet is roughly triangular in functional ventral view (posterior view in evolutionary terms); the tip is needle-like and slightly curves inwards (medially). Post-ocular segment 5 recognisable by its pair of appendages. These appendages are proximally conjoined forming a labium (maxillae in more neutral arthropod terminology). The proximal, conjoined part of the labium (mentum(?), prementum (?)) is roughly rectangular in functional ventral view (posterior), but has a distinct notch at the posterior (proximal) edge. Arising from the conjoined proximal region of the labium is a pair of non-conjoined parts of the appendages, the labial palps. None of the palps appear to be subdivided. The overall shape of the labial palp resembles the shape of the club-shaped antenna element including the tip, which, unlike in the antenna, is not separated from the more proximal region here.

The transition region from the head to the trunk forms a distinct sclerite, the cervix. The cervix is less wide than the head capsule and slightly less than half of the length of the head capsule.

#### Anterior trunk, thorax

All three segments sub-similar. Each segment about as long as cervix. Thorax segment one (post-ocular segment 6) slightly wider than cervix. Thorax segments two (post-ocular segment 7) and three (post-ocular segment 8) consecutively slightly wider. Each segment with a pair of walking appendages that are subsimilar. Each appendage is composed of at least four distinct elements. Proximal element, coxa (not equivalent to coxa in other crustaceans), short, slightly conical, tapering distally, about as long as wide at the base. Element 2, trochanter, significantly shorter and thinner, slightly less than half as wide as the proximal edge of the coxa; about as long as wide. Element 3, femur, more elongate, tube-shaped. About the same diameter as element 2, but more than three times as long. Element 4 longer than elements 2 and 3 together, slightly wider and not entirely straight. Most likely the element is either further subdivided (into tibia and tarsus), or will be further subdivided during ontogeny. Distally a pair of small curved claws is apparent (Fig. 2c).

#### Posterior trunk, abdomen

Ten distinct segments apparent. Anterior nine most likely true segments (post-ocular segments 9–17); last one most likely representing post-ocular segments 10 and 11. Abdominal segments 1–9 all of more or less equal length, consecutively narrower towards the posterior. Abdominal segment 10 about as wide as segment 9, but twice as long, rounded posteriorly. Abdominal segments 9 and 10 with distinct setae, most likely originally setae on all abdominal segments.

## Discussion

### Identity of the larval specimen

**Fig. 3:**
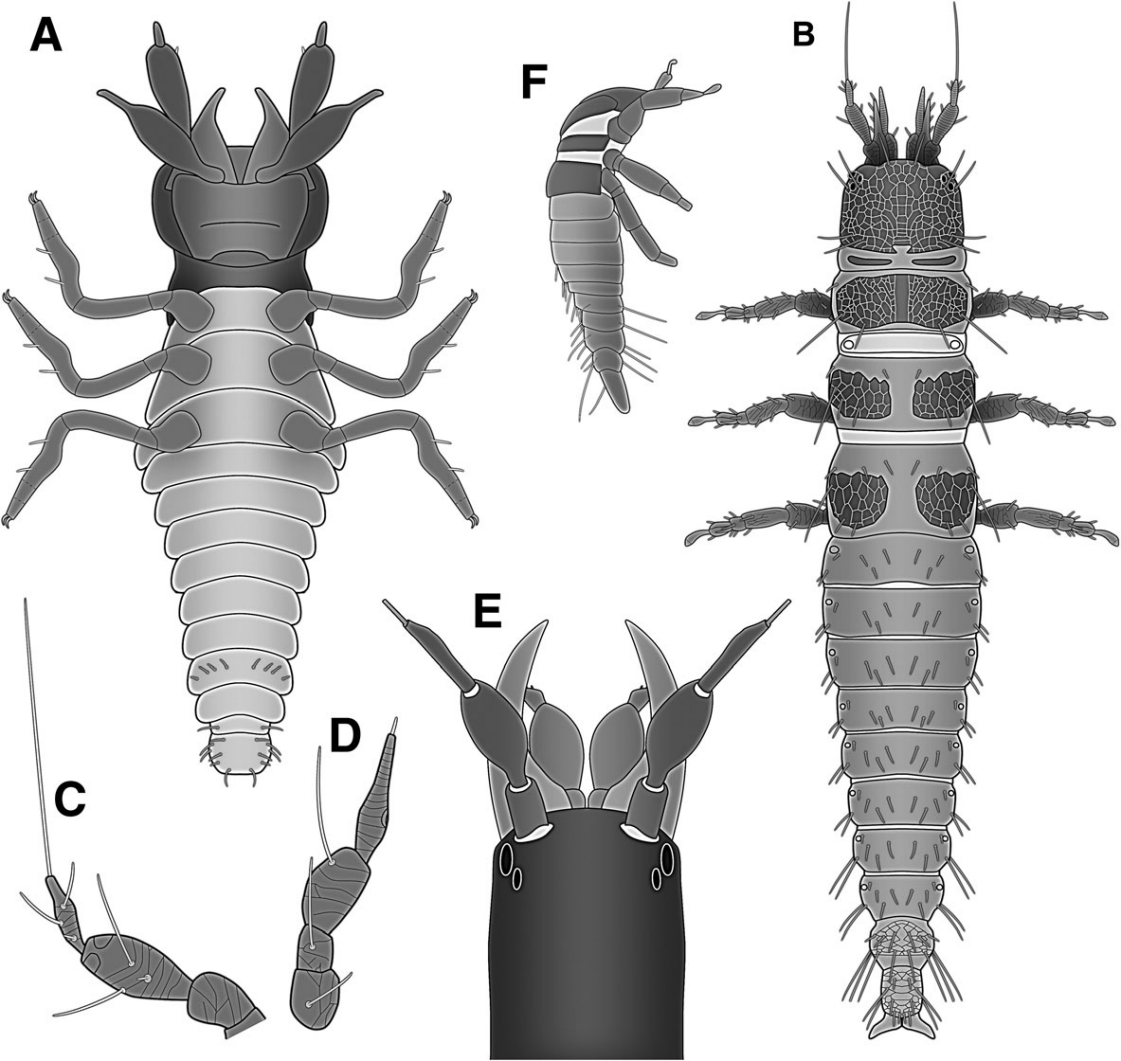
Restoration of the fossil mantis lacewing larva and comparative material of different representatives of Mantispidae. **a** Restoration of the new fossil larva in ventral view. **b** First larval stage of the extant species *Mantispa uhleri*; simplified from [5] (their Fig. 4A). **c**, **d**. Antenna (**c**) and labial palp (**d**) of first larval stage of the extant species *Mantispa pulchella*; simplified from [14] (their Fig. 15). **e** Head of *Plega melitomae*; note the curved mandibles; modified after [15] (his Fig. 42). **f** Fossil mantis lacewing larva described by Ohl [1]; simplified from [1] (his Fig. 1b)

The specimen can be easily identified as a larval representative of neuropteran insects. The overall body organisation (Fig. 3a) with three pairs of walking appendages leaves no doubt that the specimen is an insect. The strongly prognathous mouth parts combined with the arrangement of the mandibles and maxillae into an apparent pair of functional stylets and the distinct sclerotic neck (cervix) are especially strong indications that the specimen is a larval neuropteran.

The close association with a spider, even clutching the spider leg with the tarsal claws immediately gives a hint that this specimen could be a representative of Mantispidae. Many modern-day first stage mantis lacewing larvae are known to climb on spiders to later on feed on the eggs within the egg sacs of the spider.

Yet what about other characters? The strongly club-shaped elements of antennae and labial palps support the interpretation that the specimen is a first larval stage of a mantis lacewing. While some larval representatives of Hemerobiidae also have similar appearing club-shaped labial palps ([13], his plate 5), the combination of club-shaped elements on antennae and labial palps seems in modern forms only present within Mantispidae ([5], their Fig. 4A, simplified in Fig. 3b; [14], their Fig. 15, partly simplified in Fig. 3c, d). Hence this morphological structure is compatible with such an interpretation of the fossil.

A more difficult character is the structure of the mandibular-maxillary stylets: they are inward curved in the fossil, but straight in most modern forms [5, 8]. Yet there are also some less well-known cases in which modern mantis lacewing larvae possess curved mandibular-maxillary stylets ([15]; simplified in Fig. 3e).

We should furthermore expect the presence of a prominent empodium (extension between the tarsal claws) in the fossil, as this structure is known from mantispids, but also from many other neuropteran larvae (e.g., Hemerobiidae, see [13], his plate 5). However, such structures are not apparent. In the well-preserved leg grasping the spider leg the empodium might be hidden behind the spider leg (see below). The secondary absence of prominent empodia also does not seem unusual within Neuroptera.

In summary, the morphology of the specimen strongly supports an interpretation of the fossil specimen as a stage one larva of a representative of Mantispidae. Further systematic interpretations are more difficult. Curved larval mandibles occur in representatives of Symphrasinae [15]. Yet, according to Liu et al. [16] Symphrasinae is either sister group to all other mantis lacewings or even non-monophyletic. In the latter case, curved larval mandibles may well represent a plesiomorphic character. Our knowledge of larval morphology and biology of most mantis lacewings is still very incomplete [8]. We will need a denser sampling of larvae before being able to resolve character evolution of larval characters.

### A case of palaeo-parasitism?

Inferring the interactions between two extinct organisms is not as simple as in extant forms, as direct observation is not possible. Nagler and Haug [17] discussed different types of information that can be used for such an enterprise.

Two different types of indications can be applied in the present case. As always, both depend on our knowledge of the extant forms of Mantispidae. Firstly, phylogenetic inference is a strong indicator here. As argued above, the morphology of the fossil larva is a clear indicator that it is a first larval stage of a mantis lacewing, and extant mantis lacewing larvae have repeatedly been characterised as parasites (see also further below on this point).

Secondly, this interpretation is further supported by the fact that the fossil larva is in direct contact with the fossil spider. Modern forms are also known to board spiders via the legs [4]. The fact that the tarsal claws are in direct contact to the leg and the empodium is not visible, hence most likely behind the leg, makes a strong case that the spider leg was indeed tightly gripped by the larva. As not all modern-day first stage mantis lacewing larvae interact with spiders the second aspect is quite important for the present case.

It therefore seems likely that the fossil behaved in a similar way to modern-day first-stage larvae of Mantispinae, boarding spiders and waiting for eggs to be produced. The only other fossil providing a comparable case was described by Ohl [1] from significantly younger Eocene amber (Fig. 3f). Yet, in this case less morphological details of the larva were available as the head region is deeply concealed between the two body regions of the spider. On the other hand, the position on the spider is more comparable to the known final position in modern larvae. In both cases there should be little doubt that the larva is a first stage of a mantis lacewing intentionally interacting with spiders, just like their modern counterpart.

Still there remains one aspect that makes it still difficult to identify these two fossil finds as cases of palaeo-parasitism. The simple question is: are modern-day larvae of mantis lacewings indeed parasites?

### Life habits of modern-day mantis lacewing larvae: The details

Classic categories such as ‘predator’ or ‘parasite’ are in fact often not easily applied to all the variety of life strategies employed by the myriads of different insect species. As possible cases of intermediate feeding strategies many wasps, dipterans or strepsipterans have been addressed as ‘parasitoids’, adult midges as ‘micro-predators’, or better temporary parasites. This demonstrates already that our coarse categories cannot properly reflect the true diversity of ecologies out there.

Consequently, the literature seems to a certain degree “undecided” how to address the feeding habits of first stage mantis lacewing larvae: Some authors call them ‘preying’ or ‘predation’ [1, 4–6, 8], in other cases other authors (or even the same ones) refer to ‘parasitism’ [1, 18, 19] or use related expressions such as ‘host’ [1, 8, 18, 19].

Obviously, a differentiated view is necessary here. Already Redborg [4] pointed out that the habit of the larvae to feed on the spider eggs cannot be interpreted as parasitism, but represents a case of predation. Indeed, the egg sac cannot be parasitised (expression for example in [1]), and also the eggs are not only parasitised, but more or less entirely consumed.

However, some aspects are still reminiscent of parasites. The emergence of (pharate) mantis lacewings from spider egg sacs [4, 5] is strongly reminiscent of parasitoid wasps emerging from their hosts. Still the process is quite different. Even more resemblance to “classic” parasitism is present in cases in which the larva mounts the spider before it has produced the egg sac. This attachment behaviour is indeed very reminiscent of the behaviour of many parasites in the strict sense. Yet, such a behaviour could also be interpreted as a kind of phoresy, but two other aspects can apparently be coupled to spider boarding possibly qualifying for parasitism in the strict sense.

Firstly, there are few reports that some larvae do not sit at the area between prosoma and opisthosoma (the pedicel), but enter the book lungs of the spider [4, 5]. Although there is no report of details this will clearly affect the spider negatively while providing positive effects, at least shelter and humidity, for the larva. Such a behaviour is also reminiscent of well-known parasites that live in the gill chambers or lungs of their hosts, such as bopyridean isopods or tongue worms (interestingly we have different types of inferences pointing to parasitic behaviour of early fossil representatives of these groups; [20–26]).

Secondly, at least some first stage mantis lacewing larvae that board spiders before they have produced egg sacs seem to bridge the time until eggs are available by piercing the spider and feeding on its haemolymph. This last point would definitely qualify for being interpreted as parasitism in the strict sense. Unfortunately, it is not fully clear [4], but is also based on not entirely direct observation. Still it seems quite likely that some first stage mantis lacewing larvae that perform spider boarding also feed on the spider’s haemolymph, and hence are parasites in the strict sense. We can only assume that both fossils, as they boarded spiders that do not yet seem to already carry egg sacs, would also have employed this strategy and indeed represent parasites. In summary, they both represent very likely cases of palaeo-parasitism.

### A case of hypermetaboly?

As pointed out above, modern-day first stage larvae of mantis lacewings feeding on spider eggs develop through a quite unusual developmental pattern: The first stage larva in fact resembles the later adult more than the two following stages. This first stage larvae, as the adults, are mobile and active, while stage two and three larvae are largely immobile. They also lack sclerotisation. The grub-like body form is most likely of advantage for gaining size. From stage two to stage three, they can increase their size by more than 300% [5], which is quite drastic for a single moult. This is at least a possible functional explanation for the grub-like morphology: there is no selective pressure for any mobility, the morphology can therefore be “adjusted” to the main function of the larva – eat and grow.

First stage larvae largely resemble the larval stages of other neuropteran insects, especially those of Berothidae, but also those of certain representatives of Hemerobiidae or Chrysopidae. Therefore, we can assume that the mobile, campodeiform appearance of the first stage larvae is an ancestral character, the grub-like morphology is a derived one.

The grub-like morphology of the stage two and three larvae could most likely only evolve after the strategy of entering egg sacs had evolved as otherwise selective pressures would probably have acted for preserving the plesiomorphic type of morphology in these stages. We therefore assume that the earliest representatives of mantis lacewings that had evolved larvae that would enter egg sacs in the first stage would not yet have possessed grub-like larval stages two and three. Only later in this lineage could this type of morphology have evolved.

In consequence, we suggest that the presence of first stage larvae that board spiders is not necessarily a direct indication of highly specialised grub-like larvae, especially as the curved mandibles in the fossil may indicate that it still lacks some of the derived characters. We therefore disagree with Ohl [1] that hypermetaboly was necessarily present already in the stem-species of Mantispinae; it might well have evolved later, within the group. Hence, we cannot be sure about the developmental pattern of the two fossil larvae.

## Conclusions

To summarise our results:

- We report here the oldest larva of a mantis lacewing and only the second report of a fossil mantis lacewing larva so far.
- We interpret this as a case of palaeo-parasitism.
- We point out that only some aspects of the behavioural ecology of mantis lacewing larvae represent parasitism and most aspects are indeed cases of predation.
- The fossils known cannot be interpreted as indicators of a hypermetabolous type of development.
